# Matrix Product State Simulations of Non-Equilibrium Steady States and Transient Heat Flows in the Two-Bath Spin-Boson Model at Finite Temperatures

**DOI:** 10.3390/e23010077

**Published:** 2021-01-06

**Authors:** Angus J. Dunnett, Alex W. Chin

**Affiliations:** Institut des NanoSciences de Paris, CNRS, Sorbonne Université, 4 Place Jussieu, 75005 Paris, France; alex.chin@insp.upmc.fr

**Keywords:** open quantum systems, tensor networks, non-equilibrium dynamics

## Abstract

Simulating the non-perturbative and non-Markovian dynamics of open quantum systems is a very challenging many body problem, due to the need to evolve both the system and its environments on an equal footing. Tensor network and matrix product states (MPS) have emerged as powerful tools for open system models, but the numerical resources required to treat finite-temperature environments grow extremely rapidly and limit their applications. In this study we use time-dependent variational evolution of MPS to explore the striking theory of Tamascelli et al. (Phys. Rev. Lett. **2019**, *123*, 090402.) that shows how finite-temperature open dynamics can be obtained from zero temperature, i.e., pure wave function, simulations. Using this approach, we produce a benchmark dataset for the dynamics of the Ohmic spin-boson model across a wide range of coupling strengths and temperatures, and also present a detailed analysis of the numerical costs of simulating non-equilibrium steady states, such as those emerging from the non-perturbative coupling of a qubit to baths at different temperatures. Despite ever-growing resource requirements, we find that converged non-perturbative results can be obtained, and we discuss a number of recent ideas and numerical techniques that should allow wide application of MPS to complex open quantum systems.

## 1. Introduction

The physics of open quantum systems (OQS) plays a critical role in almost all aspects of quantum science [1,2], and the emergent phenomena of dephasing, decoherence and dissipation particularly limit our ability to initialise and control multi-partite quantum states. As a direct result of this, the development of scalable quantum technologies is greatly constrained by open system phenomena, and understanding how irreversibility arises from microscopic system-environment interactions has become essential for finding ways to mitigate deleterious noise effects [3]. However, alongside this goal of suppressing dissipative noise—normally by making the systems less ‘open’—the theory of OQS also plays a vital role in the design of systems where the exploitation of strong energy and information exchange between a system and its environment is desirable: this is the world of quantum thermodynamics and nanoscale energy harvesting, storage and transduction [4,5,6].

Any ‘machine’ or device capable of converting ambient energy into work must necessarily be an open system. As these machines shrink to lengths where such energetic transformations can become few-quanta, ultra-fast events, it becomes necessary to describe their functional dynamics on timescales over which system-environment correlations—in both space and time—may be highly relevant [7,8]. Unlike the perturbative OQS found, for example, in atomic systems where dissipation can be characterised by simple decay rates, quantum energy harvesting naturally focuses on the highly non-Markovian and non-perturbative regime of OQS where the border between the ‘system’ and ‘environment’ degrees of freedom is ill-defined. Moreover, as systems capable of converting thermal energy must also reject a certain amount of heat to a colder reservoir [6], the study of quantum energy harvesting leads directly to a consideration of multi-environment OQS, and the extended, inter-environmental quantum correlations that could be generated under non-equilibrium operating conditions.

Molecular and biological light-harvesting systems provide a good example of such nanoscale energy extraction, in which a non-thermal population of electronic excitations (excitons, charge pairs, etc.) appears from the molecule-mediated connection of the ‘hot’ photon and ‘cold’ vibrational environments. In this context, much attention has been placed on the complex physics due to the strong coupling and non-separable timescales of electronic and environmental (vibrational) dynamics [9,10,11], which include potentially exploitable effects such as transient breaking of detailed balance [12], noise-induced electronic coherence and cooperative multi-environment effects [13,14]. In such studies, the effect of light is normally assumed to be weak, leading to the ‘additive’ approximation that phenomenological terms describing excitation, emission and dephasing can be simply added to the more complex equations of motion of the vibronic open system. However, organic molecules often have very strong light-matter coupling and can show surprising non-additive effects [15,16], including nonlinear polaritonic weakening of exciton-phonon coupling in micro-cavity systems [17].

The example above highlights the theoretical challenges posed by some energy harvesting systems: non-perturbative and highly structured couplings, comparable dynamical timescales and competing environmental processes. Under these conditions the dissipative dynamics of the system’s reduced density matrix cannot be simply described by dephasing and relaxation rates: the full real-time evolution of the system and its environments must be accounted for on an essentially equal footing. This looks, a priori, like a hopeless task, as each environment contains a continuum of quantum excitation modes, and the formal number of quantum states in any computation will explode exponentially with the number of such modes. However, things are not so desperate, and two broad responses to this problem have emerged over recent years: one branch aims to efficiently simulate the propagators of the system’s reduced density matrix [1,9,18,19], the other aims at representing and evolving the entire system-environment wave function. Important contributions in this latter domain are Density Matrix Renormalization Group (DMRG) techniques such as the Time Evolving Density operator with Orthogonal Polynomials Approach (TEDOPA) [12,20], Dissipation-Assisted Matrix Product Factorization [21], Time-Dependent Numerical Renormalisation Group techniques and the Multi-Layer Multi Configurational Time-Dependent Hartree method (ML-MCTDH) developed in chemical physics [22,23].

The key to all of the wave function methods is the observation that, given a well-defined initial condition, the quantum dynamics generated by typical system-environment Hamiltonians leave the state inside a much smaller sub-space of the complete Hilbert space of the problem. This suggests that the wave function can be parameterised by a potentially tractable number of parameters, and—as we shall see—the effectively short-range, one-dimensional structure of OQS Hamiltonians implies that Matrix Product States (MPS) will provide an efficient and versatile format for many system-environment wave functions. Viewed this way, the parameters (matrices) of an MPS can be considered as variational degrees of freedom, leading to the powerful 1-site time-dependent variational principle (1TDVP) algorithm for efficient propagation of large wave functions in real-time [24]. This general technique can be used in any MPS and Tree-Tensor Network problem [14], but its particular utility in open-system problems has only recently been appreciated. We shall make use of this technique in this article, but a discussion of MPS, TDVP and other computational aspects is left to the dedicated presentations in the literature [24,25,26,27].

Instead, the key issue that we wish to explore in this study is the remarkable recent result of Tamascelli et al. [28] that allows wave function approaches to OQS to effectively capture the effects of finite temperature environments through the simulation of an equivalent zero-temperature proxy system. As already discussed above, in the non-perturbative, non-Markovian regime of OQS, computing the evolution of the single wave function from a sharp initial condition can already be very demanding: converging results over the astronomically large space of initial conditions in a thermal ensemble rapidly becomes impossible. If we also wish to explore the role of non-classical effects in heat flows between finite-temperature environments, the problem becomes exponentially worse. The access to finite temperature properties from a single zero-temperature (pure) wave function simulation thus opens up an entire class of powerful non-perturbative methods for the study of novel open quantum systems. This work aims to establish the extent to which Tamascelli’s ‘T-TEDOPA’ theory translates into affordable non-perturbative TDVP simulations of thermal and non-equilibrium OQS dynamics, as well as to explore some of the non-classical and non-additive aspects of heat exchange in OQS.

This article is organised as follows. In Section 2.1 we present the spin-boson Hamiltonians that we will simulate. Section 2.2 and Section 2.3 give a summary of the T-TEDOPA theory that we will employ in our numerical investigations. Section 2.4 then presents a careful study of the non-perturbative spin-boson model at finite temperatures which reveals some of the practical numerical costs implicit in this approach. Thanks to this testing, we are able to offer a freely accessible dataset that can be used as a benchmark for other numerical approaches to this model, as well as code packages that allow users to perform their own TDVP calculations on finite-temperature open systems. In anticipation of the need to explore non-equilibrium states in a wide range of future contexts, we go on to test the non-perturbative physics of a two-level system (TLS) coupled to two environments at different temperatures in Section 2.5. Exploiting the information in the many-body system-environment(s) wave function, we examine the microscopic behaviour of the heat flows between the system and the environments as a function of environmental coupling strength and temperature differences, and highlight a number of non-additive effects arising from non-perturbative quantum polaron effects. Finally, we summarise and discuss our findings in Section 3.

## 2. Results

### 2.1. Model, Parameters and Initial Conditions

**Figure 1:**
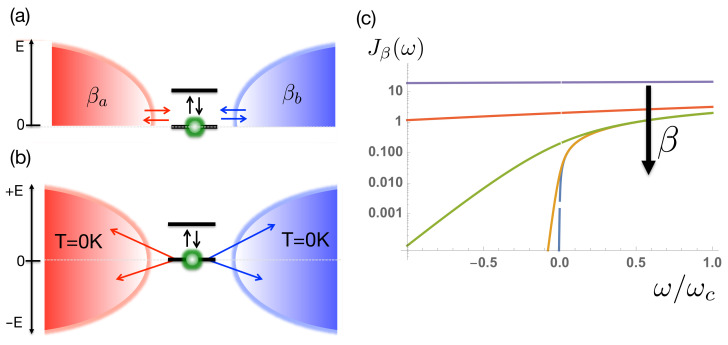
(**a**) Two-level system (TLS) is coupled to two environments (a,b) with inverse temperatures $\beta_{a}$ and $\beta_{b}$. (**b**) The reduced state dynamics of the TLS can be obtained from a zero-temperature simulation of an extended environment containing negative frequency excitation modes and temperature-dependent couplings. (**c**) The effective spectral density $J_{\beta }\left(\omega \right)$ encodes the principle of detailed balance for absorption and emission of quanta between thermal transitions in the TLS. For the Ohmic spectral density considered in this article, $J_{\beta }\left(\omega \right)$ becomes flat over the entire range $\left[-\omega_{c},\omega_{c}\right]$ as the temperature increases (β decreases). The plots shown are for $\omega_{c}\beta =0.1$ (Purple), $\omega_{c}\beta =1$ (Red), $\omega_{c}\beta =10$ (Green), $\omega_{c}\beta =50$ (Yellow) and $\omega_{c}\beta =100$ (Blue).

We shall base our exploration of finite temperature open dynamics on numerical simulations and analysis of a quantum two-level system that is strongly coupled to either one or two baths of bosonic harmonic oscillators, as illustrated in Figure 1a. The two baths are labelled *a* and *b* and are at different inverse temperatures $\beta_{a}$ and $\beta_{b}$, respectively ($\beta =1/(k_{b}T)$). The system-bath Hamiltonian is given by

(1) $$\hat{H}=\frac{\omega_{0}}{2}\sigma_{z}+{\hat{H}}_{I}^{a}+{\hat{H}}_{I}^{b}+{\hat{H}}_{B}^{a}+{\hat{H}}_{B}^{b},$$

where

(2) $$\begin{array}{cc} \; & {\hat{H}}_{I}^{a}=\sigma_{x}⊗\sum_{k}\left(g_{k}^{*}{\hat{a}}_{k}+g_{k}{\hat{a}}_{k}^{†}\right) \end{array}$$

(3) $$\begin{array}{cc} \; & {\hat{H}}_{I}^{b}=\sigma_{x}⊗\sum_{k}\left(g_{k}^{*}{\hat{b}}_{k}+g_{k}{\hat{b}}_{k}^{†}\right) \end{array}$$

(4) $$\begin{array}{cc} \; & {\hat{H}}_{B}^{a}=\sum_{k}\omega_{k}{\hat{a}}_{k}^{†}{\hat{a}}_{k} \end{array}$$

(5) $$\begin{array}{cc} \; & {\hat{H}}_{B}^{b}=\sum_{k}\omega_{k}{\hat{b}}_{k}^{†}{\hat{b}}_{k}. \end{array}$$

The TLS is described by the standard Pauli operators σ, while the ${\hat{a}}_{k}\left({\hat{b}}_{k}\right)$ are bosonic anihilation operators for harmonic modes of frequency $\omega_{k}$ in bath a(b). The corresponding creation operators are denoted ${\hat{a}}_{k}^{†}\left({\hat{b}}_{k}^{†}\right)$. The *k* harmonic of each bath couples to the TLS with a coupling strength denoted $g_{k}$, which we take to depend on the index *k* but not on *a* or *b*.

The spectral density of the environment is defined as $J\left(\omega \right)\equiv \pi \sum_{k}{\left|g_{k}\right|}^{2}\delta \left(\omega -\omega_{k}\right)$, where δ(x) is the Heaviside Theta function. As a smooth, continuous function of frequency, the spectral density can take various forms in specific physical realisations such as electron-phonon interactions, emitter-photon or exciton-vibration coupling in molecular systems. It is well known that the qualitative behaviour of the TLS depends sensitively on the form of J(ω), especially at low temperatures [1]. For simplicity, we assume identical system-bath couplings for both environments and use the common linear frequency dependence that defines an Ohmic environment

(6) $$J\left(\omega \right)=2\pi \alpha \omega \theta \left(\omega_{c}-\omega \right),$$

where α is a dimensionless coupling constant and we have introduced a hard frequency cut-off $\omega_{c}$.

The initial condition $\hat{\rho }\left(0\right)$ for our numerical simulations is taken to be an uncorrelated (product) state of the spin and baths, which—because of the baths’ finite temperatures—must be described by a mixed state, i.e., a density matrix

(7) $$\hat{\rho }\left(0\right)=\rho_{s}⊗\frac{e^{-{\hat{H}}_{B}^{a}\beta^{a}}}{\mathrm{Tr}\{e^{-{\hat{H}}_{B}^{a}\beta^{a}}\}}⊗\frac{e^{-{\hat{H}}_{B}^{b}\beta^{b}}}{\mathrm{Tr}\{e^{-{\hat{H}}_{B}^{b}\beta^{b}}\}},$$

where $\rho_{s}$ is some arbitrary initial density matrix for the TLS.

Remarkably, despite the initial condition containing two statistically mixed thermal density matrices, it has recently been shown by Tamascelli et al. that the reduced dynamics of the spin can still be obtained from a single simulation of an equivalent pure, i.e., zero temperature, system-environment wave function [28,29]. As this result is central for generating our numerical results and our later discussion, we shall now give a brief summary of the protocol first presented in Ref. [28].

### 2.2. Finite-Temperature Reduced Dynamics from Pure Wave Function Evolution

In this section we shall closely follow the original notation and presentation of Tamascelli et al. [28] and, to simplify the presentation, we shall only consider the coupling to a single environment denoted *E*. The procedure can be easily generalised to multiple environments. Our starting point is the generic Hamiltonian for a system coupled to a bosonic environment consisting of a continuum of harmonic oscillators

(8) $$H_{SE}=H_{S}+H_{E}+H_{I},$$

where

(9) $$H_{I}=A_{S}⊗\int_{0}^{\infty }d\omega {\hat{O}}_{\omega },H_{E}=\int_{0}^{\infty }d\omega \omega a_{\omega }^{†}a_{\omega }.$$

The Hamiltonian $H_{S}$ is the free system Hamiltonian and $A_{S}$ is a generic system operator which couples to the bath. The environment’s free Hamiltonian is given by $H_{E}$. For the bosonic bath operators we take the displacements

(10) $$O_{\omega }=\sqrt{J(\omega )}\left(a_{\omega }+a_{\omega }^{†}\right),$$

thus defining the spectral density J(ω). This has been written here as an arbitrary continuous function, but we note that the formulas can also be applied to the case of coupling to a discrete set of vibrational modes by adding suitable structure to the spectral density, i.e., sets of lorentzian peaks or Dirac functions [30,31,32].

The state of the system+environment at time *t* is a mixed state described by a density matrix $\rho_{SE}\left(t\right)$. The initial condition is assumed to be a product of system and environment states $\rho_{SE}\left(0\right)=\rho_{S}\left(0\right)⊗\rho_{E}\left(0\right)$ where $\rho_{S}\left(0\right)$ is an arbitrary density matrix for the system and $\rho_{E}\left(0\right)=\exp\left(-H_{E}\beta \right)/Z$, with the environment partition function given by $Z=\mathrm{Tr}\{\exp\left(-H_{E}\beta \right)\}$. Such a product state is commonly realised in non-equilibrium problems where the system is suddenly prepared or projected into an excited state from a ground state in which the system and environment states are separable. This type of preparation is exemplified by the Franck-Condon principle in molecular photophysics, where the optical transition occurs without any change in the nuclear degrees of freedom, leaving the subsequent relaxation dynamics to evolve from a product ‘initial’ condition [33,34]. The environment thus begins in a thermal equilibrium state with inverse temperature β, and the energy levels of each harmonic mode are statistically populated. For a very large number (continuum) of modes, the number of possible thermal configurations grows extremely rapidly with temperature, essentially making it impossible to obtain a converged sampling of these configurations when each instance involves demanding wave function simulations. We briefly note that some more efficient sampling methods involving sparse grids and/or stochastic mean-field approaches have recently been proposed and demonstrated [35,36], as well as some effective MPS techniques for capturing finite temperature effects in frequency domain simulations [37].

The initial thermal condition of the environmental oscillators is also a Gaussian state, for which it is further known that the influence functional [1]—which is a full description of the influence of the bath on the system—will depend only on the two-time correlation function of the bath operators

(11) $$S\left(t\right)=\int_{0}^{\infty }d\omega \langle O_{\omega }\left(t\right)O_{\omega }\left(0\right)\rangle .$$

Any two environments with the same S(t) will have the same influence functional and thus give rise to the same reduced system dynamics, i.e., the same $\rho_{S}\left(t\right)=\mathrm{Tr}\left\{\rho_{SE}\left(t\right)\right\}$. That the reduced density matrix’s dynamics are completely specified by the spectral density and temperature of a Gaussian environment has been known for a long time [1], but the key idea of the equivalence—and thus the possibility of the interchange—of environments with the same correlation functions has only recently been demonstrated by Tamascelli et al. [29].

The time dependence in Equation (11) refers to the interaction picture so that the bath operators evolve under the free bath Hamiltonian: $O_{\omega }\left(t\right)=e^{iH_{E}t}O_{\omega }\left(0\right)e^{-iH_{E}t}$. Using Equation (10) and $\langle a_{\omega }^{†}a_{\omega }\rangle =n_{\beta }\left(\omega \right)$ we have

(12) $$S\left(t\right)=\int_{0}^{\infty }J\left(\omega \right)\left[e^{-i\omega t}\left(1+n_{\beta }\left(\omega \right)\right)+e^{i\omega t}n_{\beta }\left(\omega \right)\right].$$

Making use of the relation

(13) $$\frac{1}{2}\left(1+\coth\left(\omega \beta /2\right)\right)\equiv \left\{\begin{array}{c} n_{\omega }\left(\beta \right),\omega \geq 0\\ -(n_{\left|\omega \right|}\left(\beta \right)+1),\omega <0 \end{array}\right.$$

we can write Equation (12) as an integral over all positive and negative ω

(14) $$S\left(t\right)=\int_{-\infty }^{\infty }d\omega \mathrm{Sign}\left(\omega \right)\frac{J(|\omega |)}{2}\left(1+\coth\left(\frac{\omega \beta }{2}\right)\right)e^{-i\omega t}.$$

However, Equation (14) is exactly the two-time correlation function one would get if the system was coupled to a bath, now containing positive and negative frequencies, at zero temperature! The effects of the finite, physical temperature now appear in a new effective spectral density for the extended environment given by

(15) $$J_{\beta }\left(\omega \right)=\mathrm{Sign}\left(\omega \right)\frac{J(|\omega |)}{2}\left(1+\coth\left(\frac{\omega \beta }{2}\right)\right).$$

Thus, we find that our open system problem is completely equivalent to the one governed by the Hamiltonian

(16) $$H=H_{S}+H_{E}^{\mathrm{ext}}+H_{I}^{\mathrm{ext}},$$

in which the system couples to an extended environment, where

(17) $$\begin{array}{cc} \; & H_{I}^{\mathrm{ext}}=A_{S}⊗\int_{-\infty }^{\infty }d\omega \sqrt{J_{\beta }\left(\omega \right)}\left(a_{\omega }+a_{\omega }^{†}\right),\\ \; & H_{E}^{\mathrm{ext}}=\int_{-\infty }^{\infty }d\omega \omega a_{\omega }^{†}a_{\omega }, \end{array}$$

and which has the initial condition $\rho_{SE}\left(0\right)=\rho_{S}\left(0\right)⊗{|0\rangle }_{E}\langle 0|$. This transformed initial condition is now far more amenable to simulation as the environment is now described by a pure, single-configuration wave function, rather than a statistical mixed state, and so no statistical sampling is required to capture the effects of temperature on the reduced dynamics!

Analysing the effective spectral density of Equation (15), it can be seen that the new extended environment has thermal detailed balance between absorption and emission processes encoded in the ratio of the coupling strengths to the positive and negative modes in the extended Hamiltonian (see Figure 1c), as opposed to the operator statistics of a thermally occupied state of the original, physical mode, i.e.

(18) $$\frac{J_{\beta }\left(\omega \right)}{J_{\beta }\left(-\omega \right)}=\frac{{\langle a_{\omega }a_{\omega }^{†}\rangle }_{\beta }}{{\langle a_{\omega }^{†}a_{\omega }\rangle }_{\beta }}=e^{\beta \omega }$$

Indeed, from the system’s point of view, there is no difference between the absorption of a quantum from a thermally occupied, positive energy bath mode and the creation (emission) of an excitation into an unoccupied, negative energy, bath mode. The extension to negative frequencies essentially allows the process whereby the system would be heated by the environment (absorbing pre-existing quanta in the thermal bath) to be mimicked by spontaneous emission into a negative energy vacuum of states, as shown in Figure 1b.

In fact, the equivalence between these two environments goes beyond the reduced system dynamics as there exists a unitary transformation which links the extended environment to the original thermal environment. This means that one is able to reverse the transformation and calculate thermal expectations for the actual bosonic bath such as ${\langle a_{\omega }^{†}\left(t\right)a_{\omega }\left(t\right)\rangle }_{\beta }$. This is particularly useful for molecular systems in which environmental (vibrational) dynamics are also important observables that report on the mechanisms and pathways of physio-chemical transformations [38,39,40]. In this article, we will use this capability later to look at the non-equilibrium heat flows between the TLS and its environments. This is a major advantage of many-body wave function approaches, as full information about the environment is available, cf. effective master equation descriptions which are obtained after averaging over the environmental state.

### 2.3. Chain Mapping and Chain Coefficients

**Figure 2:**
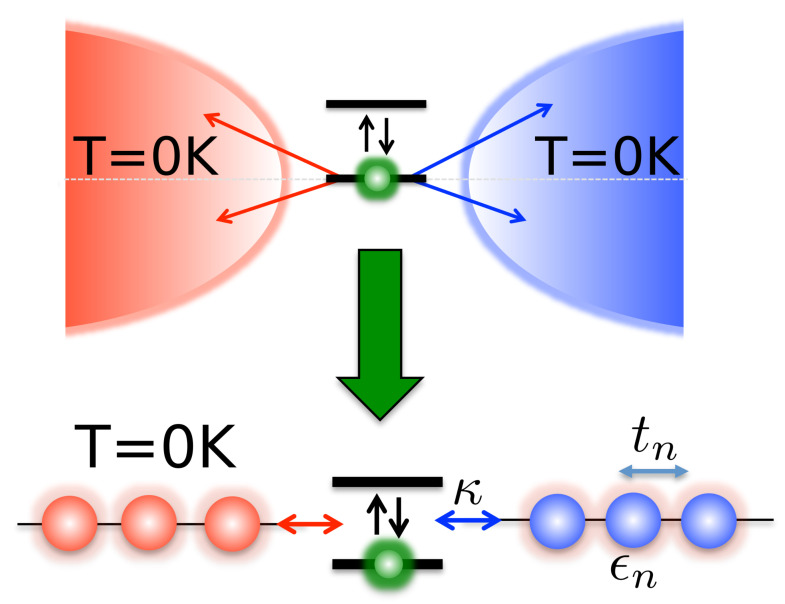
The positive and negative energy modes of each extended environment are mapped onto 1D chains with nearest-neighbour hopping, each coupled by their first site to the TLS with coupling strength κ. The chain parameters $\epsilon_{n}$ and $t_{n}$ are determined such that the eigen-modes of the chains are the original modes of the extended environments. The 1D geometry of the transformed system and the fact that the chain modes all start in their vacuum states, means the system-environment state can be described by a single (pure) matrix product states (MPS).

Following this transformation a further step is required to facilitate efficient simulation of the many-body system+environment wave-function. This is to apply a unitary transformation to the bath modes which converts the star-like geometry of $H_{I}^{\mathrm{ext}}$ into a chain-like geometry, thus allowing the use of Matrix-Product-State (MPS) methods [10,41,42] (see Figure 2). We thus define new modes $c_{n}^{\left(†\right)}=\int_{-\infty }^{\infty }U_{n}\left(\omega \right)a_{\omega }^{\left(†\right)}$, known as chain modes, via the unitary transformation $U_{n}\left(\omega \right)=\sqrt{J_{\beta }\left(\omega \right)}p_{n}\left(\omega \right)$ where $p_{n}\left(\omega \right)$ are orthonormal polynomials with respect to the measure $d\omega J_{\beta }\left(\omega \right)$. Thanks to the three term recurrence relations associated with all orthonormal polynomials $p_{n}\left(\omega \right)$ [41], only one of these new modes, n=1, will be coupled to the system, while all other chain modes will be coupled only to their nearest neighbours [41]. Our interaction and bath Hamiltonians thus become

(19) $$\begin{array}{cc} \; & H_{I}^{\mathrm{chain}}=\kappa A_{S}\left(c_{1}+c_{1}^{†}\right),\\ \; & H_{E}^{\mathrm{chain}}=\sum_{n=1}^{\infty }\epsilon_{n}c_{n}^{†}c_{n}+\sum_{n=1}^{\infty }\left(t_{n}c_{n}^{†}c_{n+1}+h.c\right). \end{array}$$

**Figure 3:**
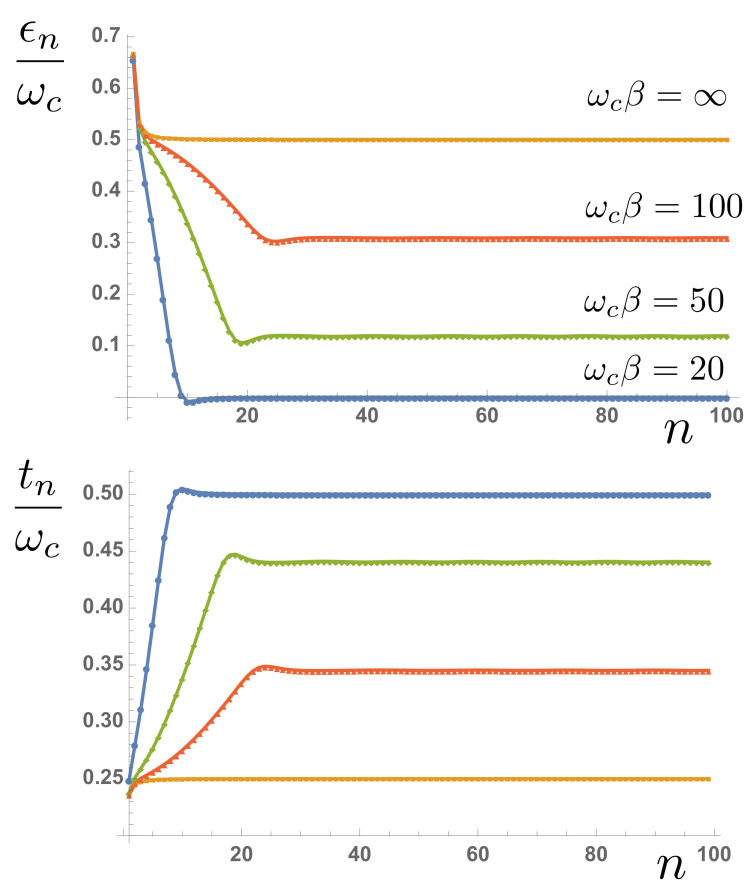
Site energies $e_{n}$ and hopping amplitudes $t_{n}$ as a function of chain distance *n* at different environment temperatures.

The chain coefficients appearing in Equation (19) are related to the three-term recurrence parameters of the orthonormal polynomials and can be computed using standard numerical techniques [41]. Since the initial state of the bath was the vacuum state, it is unaffected by the chain transformation. We briefly note the evolution of the asymptotic values of the chain parameters, as illustrated in Figure 3. For a smooth spectral density with a hard cut-off, it is has been rigorously proven that $\epsilon_{n}\to \omega_{c}/2,t_{n}\to \omega_{c}/4$ as n→∞ [41]. Figure 3 shows the dramatic changes in these asymptotic values as the temperature is increased, which—from the numerical results—appear to be $\epsilon_{n}\to 0,t_{n}\to \omega_{c}/2$ as n→∞ and β→0. This can be naturally understood from the behaviour of the effective spectral functions $J_{\beta }\left(\omega \right)$ with increasing temperature, as illustrated in Figure 1c. The spectral functions become symmetric and have finite values over the whole domain $\left[-\omega_{c},\omega_{c}\right]$. The asymptotic spectrum of the chain modes thus has a bandwidth of $2\omega_{c}$ centred on ω=0, which, for a uniform hopping chain, requires the numerically observed asymptotic chain parameters. In the particular case of the Ohmic environment at high temperatures, it can easily be seen that $J_{\beta }\left(\omega \right)$ tends to a constant and so will have a chain representation derived from the classical Legendre polynomials [41].

We have thus arrived at a formulation of the problem of finite-temperature open systems in which the many-body environmental state is initialised as a pure product of trivial ground states, whilst the effects of thermal fluctuations and populations are encoded in the Hamiltonian chain parameters and system-chain coupling. These parameters must be determined once for each temperature but—in principle—the actual simulation of the many-body dynamics is now no more complex than a zero-temperature simulation!

### 2.4. Spin-Boson Model Across the Complete α−β Space

In this section, we numerically verify that the finite-temperature approach set out in Section 2.2 and Section 2.3 captures the correct non-perturbative behaviour in the single-bath spin-boson model. This will be illustrated with a few explicit examples, but the key result of this section is the creation of a comprehensive dataset for the Ohmic spin-boson model that allows arbitrary TLS initial conditions to be propagated in real-time and over a large area of α−β space. This dataset has been made freely available online in citable form and can be used to benchmark other methods and applications [43].

**Figure 4:**
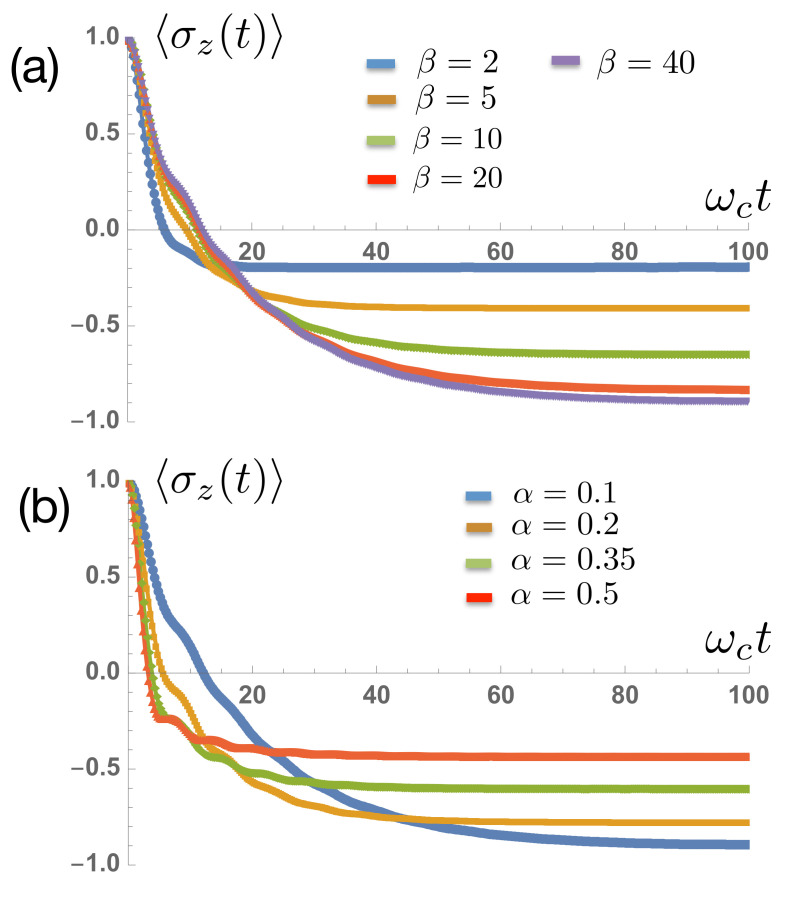
Relaxation of spin polarization as a function of time for (**a**) different temperatures and α=0.1 and (**b**) different coupling strengths with a fixed $\omega_{c}\beta =100$.

Figure 4a,b shows the temporal decay of an initially polarised spin $\langle \sigma_{z}\left(0\right)\rangle =+1$ towards thermal equilibrium for varying coupling strengths α and inverse temperatures β. The TLS energy splitting was $\omega_{0}=0.2\omega_{c}$. The key result in Figure 4b is the dependence of the thermalized spin polarization at long times. In a simple, perturbative rate equation treatment, this final polarization would be set by the energy gap $\omega_{0}$ and the temperature, according to the Gibbs-Boltzmann distribution

(20) $${\langle \sigma_{z}\rangle }_{\beta }=-\frac{\left(1-e^{-\beta \omega_{0}}\right)}{\left(1+e^{-\beta \omega_{0}}\right)}.$$

The coupling strength α would only alter the rate at which this thermal distribution is reached. However, Figure 4a shows a growing dependence of the final polarization on the coupling strength, suggesting a non-perturbative effect. This is indeed the case: strong coupling leads to polaron formation and non-perturbative renormalisation of the TLS energy gap $\omega_{0}$. According to the variational theory of Silbey and Harris [44], the renormalized gap $\omega_{r}$ is approximately given by

(21) $$\omega_{r}=\omega_{0}{\left(\frac{\omega_{0}}{\omega_{c}}\right)}^{\frac{\alpha }{1-\alpha }},$$

in the so-called scaling limit in which $\omega_{c}$ is much larger than all other energy scales in the problem. This renormalisation is highly non-perturbative, and can completely close the TLS energy gap at a critical coupling $\alpha_{c}=1$ [1]. Replacing $\omega_{0}$ with the the renormalized energy gaps in Equation (20), ${\langle \sigma_{z}\rangle }_{\beta }$ is given

(22) $${\langle \sigma_{z}\rangle }_{\beta }=-{\left(\frac{\omega_{0}}{\omega_{c}}\right)}^{\frac{\alpha }{1-\alpha }}\frac{\left(1-e^{-\beta \omega_{r}}\right)}{\left(1+e^{-\beta \omega_{r}}\right)}.$$

where the prefactor in Equation (22) accounts for the suppressed expectation values of $\sigma_{z}$ in the polaronic eigenbasis.

**Figure 5:**
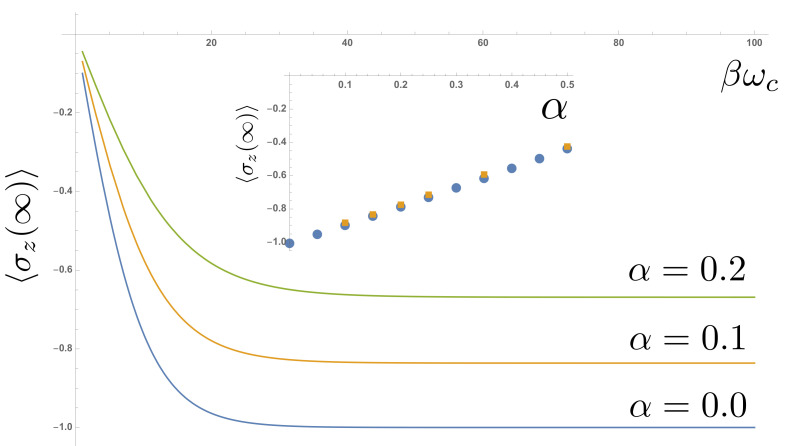
Analytical prediction of thermal steady state spin polarization as a function of inverse temperature $\omega_{c}\beta$. Inset compares these predictions with steady state values extracted from the real-time dynamics shown in Figure 4. A re-scaled coupling strength $\tilde{\alpha }=c\alpha$ with c=0.66 has been applied when evaluating the anaytical formula (see main text).

Figure 5 shows this analytical prediction as a function of temperature, compared to the results extracted from the real-time dynamics. As mentioned above, most analytical predictions for the SBM are obtained deep in the scaling limit, while numerical results necessarily involve only moderately large values of $\omega_{c}$. When comparing results, it is common in the literature to evaluate analytical expressions with a re-scaled coupling strength $\tilde{\alpha }=c\alpha$ to account for this [45,46,47], which we have applied in Figure 5. We found that a constant factor c=0.66 gave excellent agreement across the parameter space for both one and two-bath SBMs, as shown in the inset of Figure 5.

**Figure 6:**
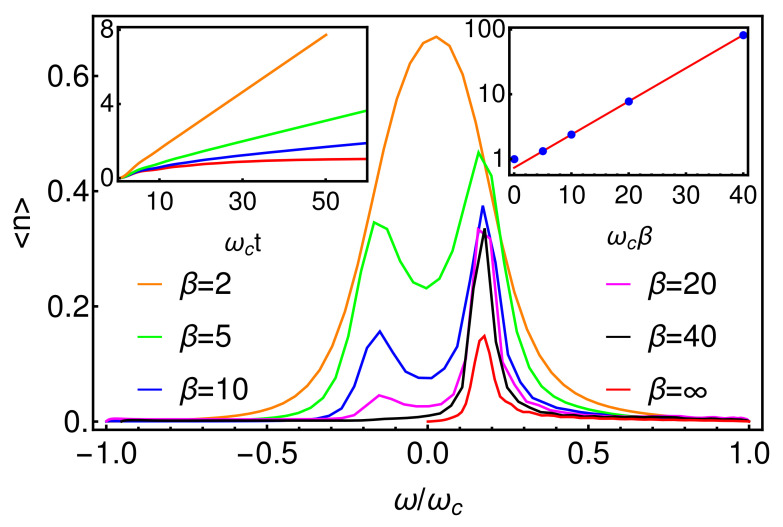
Long-time occupations of the modes of the extended environment, following the thermalization of the TLS at various temperatures. Inset shows the total number of quanta in the environment as a function of time (left) for different temperatures. This population grows indefinitely at finite temperatures. The inset (right) shows the long-time ratio of the peak heights in each curve of the main figure. These give a very good fit to the exponential dependence expected for absorption and emission rates obeying detailed balance.

As a final set of observations in this section, we now look at the behaviour of the environment. In Figure 6 we present the occupations of the bath modes in the extended spectral representation used to account for finite temperatures. As anticipated in our discussion in Section 2.2, we find that at low temperatures, the energy released from the decay of the spin is absorbed by modes with positive frequencies matching the TLS energy gap $\omega_{0}$. As the temperature increases, peaks appear at negative frequencies, corresponding to the excitation of these modes due to ‘heating’ of the TLS, i.e., the TLS is thermally excited and removes energy from the environment. As a function of temperature, the ratio of the positive and negative occupations is a very close fit to $e^{\beta \omega_{0}}$, as expected from detailed balance. However, due to the presence of negative frequency modes, we find that the populations in both the positive and negative frequency modes grow indefinitely during the simulation time, as shown in the inset of Figure 6. The difference of these growing populations plateaus at a finite value, corresponding to the thermal occupation of the physical positive-frequency mode, but care must be taken to get converged results for long-time (steady state) quantities due to the expanding local Hilbert spaces needed for the environment modes in the simulations.

**Figure 7:**
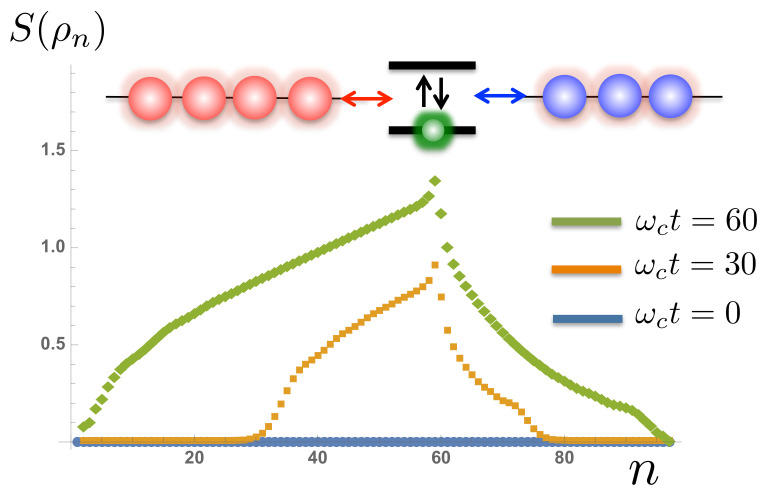
The time evolution of the von Neumann entanglement entropy for each bi-partition of the system-environment chain at site *n*. The TLS is located in this example at site n=59, with the hot bath corresponding to sites 1−58 and remaining sites representing the cold bath.

Figure 7 shows the behaviour of the von-Neumann entropy obtained by bi-partitioning the total *N*-site system-environment chain into chains of size *n* and N−n and computing the singular values of either of the subsystems’ reduced density matrices [48]. The entanglement entropy directly reports on the size of the bond-dimensions required to represent the state accurately in the MPS format, and our results show that this entropy also grows continuously during the simulation. There is also a clear asymmetry in the rate of spreading and magnitudes of entanglement, with correlations between sites in the hot environment growing much faster. Again, these growing numerical resources for finite temperature simulations should be handled with care, and we shall take this up again in Section 3.

### 2.5. Non-Equilibrium Heat Flows

In this section we simulate the non-equilibrium dynamics of the TLS connected to two environments at different temperatures. For clarity we will designate environment *a* as the ‘hot’ environment and *b* as the ‘cold’ one, using suffixes ‘h’ and ‘c’, respectively. We note here that this elementary class of two-environment models has both wide-ranging practical applications—such as studying heat and charge transfer in nano-devices and molecules [6,49,50], as well as being of fundamental relevance for quantum thermodynamics, decoherence, and non-equilibrium steady states [47,51,52,53,54].

**Figure 8:**
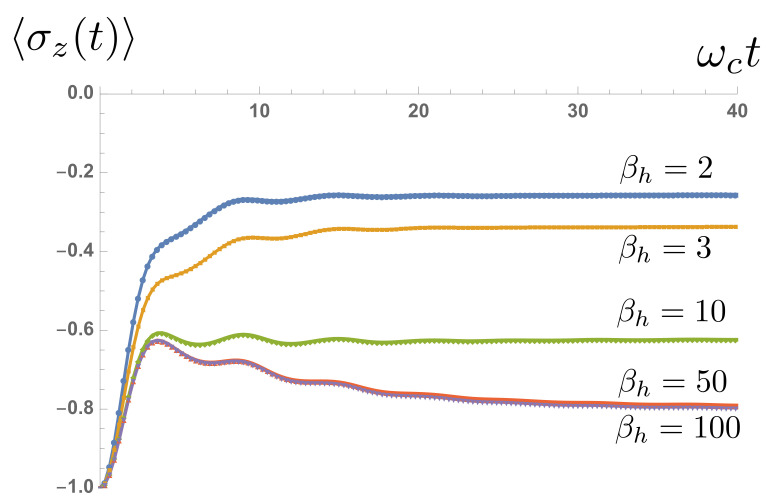
Non-equilibrium relaxation of spin polarization as a function of time for fixed cold bath temperature and varying hot bath temperatures.

**Figure 9:**
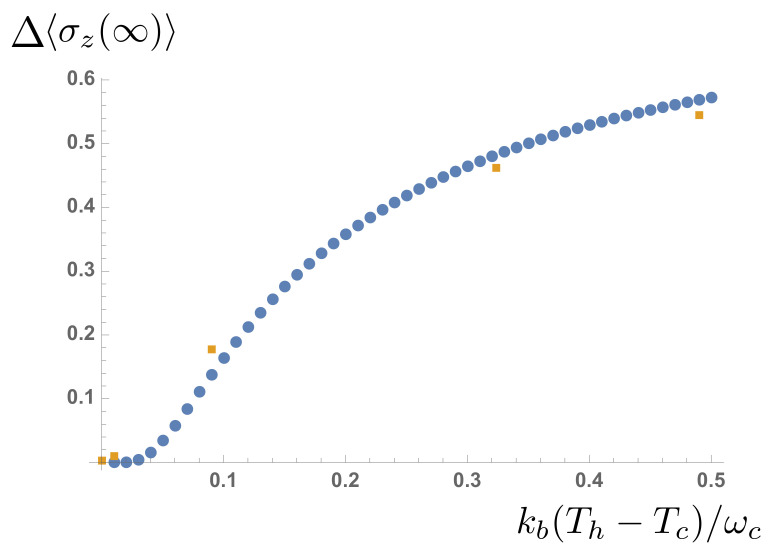
The change in $\langle \sigma_{z}\left(\infty \right)\rangle$ as a function of the temperature difference between the baths ($T_{c}$ is kept constant). Analytical predictions are shown as dots, numerical data points as squares.

Figure 8 shows the real-time excitation of a TLS initially prepared in its ground state when connected at t=0 to the cold environment with fixed $\omega_{0}\beta_{c}=100$ and the ‘hot’ environment at different temperatures. Figure 9 shows the steady-state spin polarization as a function of the temperature difference between the hot and cold baths. To understand the basic features of the steady state, let’s consider a perturbative set of rate equations for the population of the spin-up level $P_{↑}\left(t\right)$. Assuming that the rates of absorption and emission from each bath of TLS obey detailed balance, the dynamics of $P_{↑}\left(t\right)$ can be obtained from the equation

(23) $$\frac{dP_{↑}\left(t\right)}{dt}=-\Gamma P_{↑}\left(t\right)\left(n_{c}+n_{h}+2\right)+\Gamma \left(1-P_{↑}\left(t\right)\right)\left(n_{c}+n_{h}\right),$$

where $n_{i}={\left[\exp\left(\omega_{0}\beta_{i}\right)-1\right]}^{-1}$ By finding the steady state population $P_{↑}\left(\infty \right)$, the non-equilibrium value of the spin polarisation $\langle \sigma_{z}^{ab}\left(\infty \right)\rangle$ then takes the simple form

(24) $$\langle \sigma_{z}^{ab}\left(\infty \right)\rangle =\frac{-1}{2(n_{c}+n_{h}+1)}.$$

Once again, if renormalization effects are included, the agreement between the analytical predictions is very good, as can be seen in Figure 9. Indeed, for the lowest temperatures, the spin dynamics are entirely due to renormalization effects, as thermal occupation of the excited level is negligible.

Interestingly, these two-bath results also reveal an intriguing non-additive effect due to the coupling to two environments. The subject of non-additivity of environmental interactions has recently attracted attention due to the role of multiple environments in a wide range of ‘active’ quantum machines, such as the conversion of ambient solar energy in room-temperature (phonon-coupled) devices [15,16,47], and also the highly co-operative actions of different types of vibrational motion in molecular photo physics [14]. In the present case, the non-additive effects appear in the polaronic renormalisation, which is mostly clearly seen in the case when the two baths have the same temperature. This situation is indistinguishable from a coupling to a single bath with twice the coupling strength. The renormalisation can thus be obtained from Equation (21) with the replacement α→2α. However, the renormalisation arises from the overlap of the displaced mode wave functions that are ‘fast’ enough to co-tunnel with the TLS as it transitions between $\langle \sigma_{x}\rangle =\pm 1$ [44,45], and in an additive approximation the renormalization would be simply be the product of the individual overlaps for each environment. However, as is clear form Equation (21), this doubling of the coupling does not lead to a simple exponential doubling of the renormalization, but instead leads to a nonlinear suppression of the energy gap according to the exponent 2α/(1−2α).

**Figure 10:**
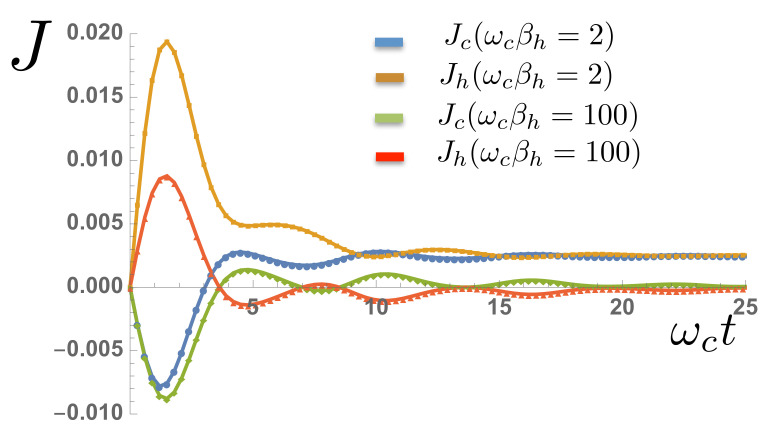
Heat flows into the cold bath ($J_{c}$) and out of the hot bath ($J_{h}$) as a function of time for varying hot bath temperatures and a fixed $\omega_{c}\beta_{c}=100$.

**Figure 11:**
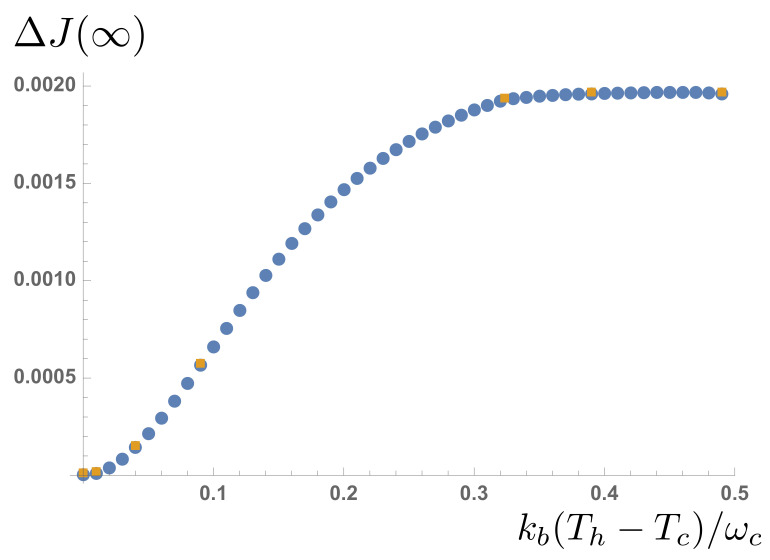
Net steady-state heat flux through the two-level system as a function of temperature difference for a fixed $\beta_{c}=100$. Data extracted from MPS simulations (yellow squares) is compared with the analytical expression in the main text (blue dots).

Exploiting the access to the environmental state, we now show the transient dynamics of the heat flow in the two baths during the establishment of the TLS steady state. We define the following operators

(25) $${\hat{J}}_{c}={\hat{\sigma }}_{y}⊗\left({\hat{A}}_{0}^{†}+{\hat{A}}_{0}\right),$$

and

(26) $${\hat{J}}_{h}={\hat{\sigma }}_{y}⊗\left({\hat{B}}_{0}^{†}+{\hat{B}}_{0}\right),$$

which measure the heat flux from the spin to baths *a* and *b* respectively. The operators ${\hat{A}}_{0}^{\left(†\right)}\left({\hat{B}}_{0}^{\left(†\right)}\right)$ refer to the creation and anihilation operators of the first site of chain a(b), i.e. the site coupled to the TLS. Representative heat flows are shown in Figure 10 for large and zero differences in the bath temperatures. In both cases, the initial dynamics involve heating from both hot and cold environments, as the spin is initially in a pure (T=0K) ground state. As the dynamical steady state of the spin is obtained, a net heat current appears from the hot to cold environment. This heat current vanishes as the temperature difference of the baths is reduced, as we would expect. From the long-time solution of the Pauli master equation given in Equation (23), the steady-state heat flux from the hot to cold environment can be shown to be

(27) $$J=\Gamma \frac{n_{h}-n_{c}}{1+n_{h}+n_{c}},$$

and this is plotted alongside our numerical data in Figure 11. The simulations correctly capture the essentially non-linear behaviour of heat flow through the quantum ‘heat leak’ TLS, although a linear regime where Fourier’s law of heat flow appears to hold can be clearly observed before the flows saturate for large temperature differences.

## 3. Discussion

The results presented in Section 2 demonstrate that accurate reduced system behaviour in the spin-boson model can be obtained in the presence of both a single or two finite-temperature environments. Non-perturbative effects related to system-bath entanglement (polaronic dressing) are captured in transient relaxation and non-equilibrium steady states, and we have shown how the T-TEDOPA transformation provides direct information related to the energy and entanglement entropy flows in the environment. All of these results were obtained from pure wave function evolution of an initial zero-temperature (vacuum) state, and the onerous numerical cost of having to sample over a thermal distribution of initial states was entirely avoided.

**Figure 12:**
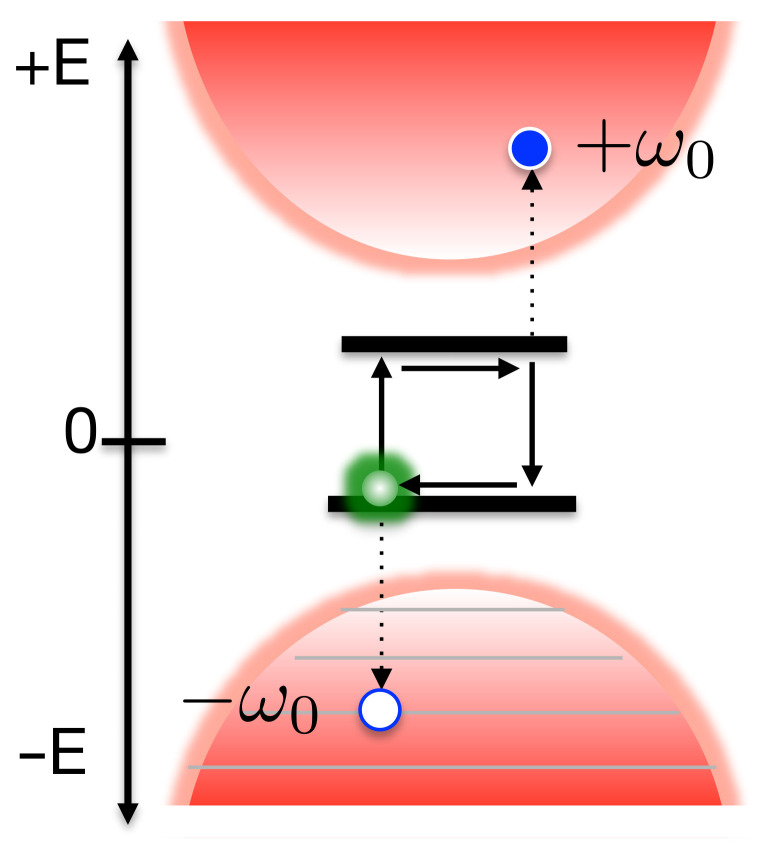
Due to the unbounded bosonic nature of the negative-frequency environment, thermal transitions within the TLS lead to a constant creation of correlated, particle-hole-like excitations in both environments with the same absolute energy $\omega_{0}$.

However, we did note that the numerical resources required to obtain these results grew in an unbounded way as a function of simulation time. In the case of the one-bath SBM, Figure 6 shows that the total number of bosonic excitation grows approximately linearly in time and the growth rate increases with the bath temperature. The main panel showing the populations of the environment in frequency space shows that this growth is the result of growing populations at frequencies $\approx \pm \omega_{0}$. In Section 2.2 we made the observation that creating an excitation in a negative frequency mode allows the TLS to be excited with overall conservation of energy, and this is the process that accounts for the ‘heating’ expected of a finite-temperature bath. The constant growth of excitations in the environment can be seen to arise from the constant cycling of the heating process sketched in Figure 12 (a similar cycle for cooling also generates a net population of excitations). Here the creation of a negative frequency excitation (or hole) excites the TLS and then is de-excited by the creation of an excitation in the positive frequency environment.

Interestingly, this pair creation goes beyond populations: the dynamics of thermalization entangles the positive and negative frequency environments. This is perhaps unsurprising in the context of the thermofield theory of De Vega et al. where the thermal entanglement properties of two-mode squeezed states are used to create an effective finite temperature environment from two zero-temperature baths [55]. However, for our open-system problem, it should be kept in mind that the ’dynamics’ of the positive and—especially—the negative modes really only provide insight into the internal workings of the simulation. The modes and their populations are proxy (non-physical) degrees of freedom used to provide vacuum fluctuations that mimic the physical interactions of the system with a strictly positive-frequency harmonic bath at finite-temperature. However, a hopefully fruitful and more physical connection between the behaviour of the artifically extended environment inT-TEDOPA can be made to very recent developments in the theory of MPS and tensor networks for fermionic quantum transport. Here, non-equilibrium particle flows between reservoirs at different chemical potentials lead to the constant creation of entangled particle-hole pairs, leading to the exponential-in-time growth of MPS bond dimensions. However, Rams and Zwolak have recently demonstrated that a change in basis used for certain fermionic transport simulations can greatly suppress the rapid growth of numerical resources [56], and it would be very interesting to see how this might translate—or might to some extent already be implemented—in our current approach to bosonic heat flow problems. Finally, we also point out that rapid growth of bond dimensions and entanglement in non-equilibrium systems is potentially a problem for 1TDVP simulations, as these proceed at fixed bond-dimensions. Choosing large bond-dimensions may allow one to reach long times, but much of the simulation is likely to run slowly, as it will be using far more resources than are necessary for most of the time. In a recent development, Dunnett and Chin have proposed an adaptive version of 1TDVP that is able to change bond-dimensions during the course of a single simulation run, allowing the necessary resources to be deployed as needed [57]. We thus conclude that recent insights and computational development have opened a whole new domain of finite and multiple-temperature open system problems for wave function techniques, and that creating numerically efficient finite-temperature simulations will inspire further progress in tensor network theory, as applied to open systems.

## 4. Materials and Methods

All numerical results were obtained using software packages that are available at https://github.com/angusdunnett/MPSDynamics. The benchmark data for the Ohmic Spin-Boson Model can be found at [43].

## Data Availability

Data sharing not applicable.
